# Intraoperative optical coherence tomography for assessing human lymph nodes for metastatic cancer

**DOI:** 10.1186/s12885-016-2194-4

**Published:** 2016-02-23

**Authors:** Ryan M. Nolan, Steven G. Adie, Marina Marjanovic, Eric J. Chaney, Fredrick A. South, Guillermo L. Monroy, Nathan D. Shemonski, Sarah J. Erickson-Bhatt, Ryan L. Shelton, Andrew J. Bower, Douglas G. Simpson, Kimberly A. Cradock, Z. George Liu, Partha S. Ray, Stephen A. Boppart

**Affiliations:** Beckman Institute for Advanced Science and Technology, University of Illinois at Urbana-Champaign (UIUC), 405 N. Mathews Ave., Urbana, IL 61801 USA; Department of Electrical and Computer Engineering, UIUC, Illinois, USA; Department of Bioengineering, UIUC, Illinois, USA; Department of Statistics, UIUC, Illinois, USA; Carle Foundation Hospital, Urbana, IL USA; Department of Surgery, University of Illinois College of Medicine at Urbana-Champaign and Carle Cancer Center, Urbana, IL USA; Department of Internal Medicine, UIUC, Illinois, USA; PhotoniCare, Inc., Champaign, IL USA; Department of Biomedical Engineering, Cornell University, Ithaca, NY USA; Carl Zeiss Meditec, Inc., Dublin, CA USA

**Keywords:** Breast cancer, Lymph node, Metastasis, Optical coherence tomography, Intraoperative

## Abstract

**Background:**

Evaluation of lymph node (LN) status is an important factor for detecting metastasis and thereby staging breast cancer. Currently utilized clinical techniques involve the surgical disruption and resection of lymphatic structure, whether nodes or axillary contents, for histological examination. While reasonably effective at detection of *macrometastasis*, the majority of the resected lymph nodes are histologically negative. Improvements need to be made to better detect *micrometastasis*, minimize or eliminate lymphatic disruption complications, and provide immediate and accurate intraoperative feedback for in vivo cancer staging to better guide surgery.

**Methods:**

We evaluated the use of optical coherence tomography (OCT), a high-resolution, real-time, label-free imaging modality for the intraoperative assessment of human LNs for metastatic disease in patients with breast cancer. We assessed the sensitivity and specificity of double-blinded trained readers who analyzed intraoperative OCT LN images for presence of metastatic disease, using co-registered post-operative histopathology as the gold standard.

**Results:**

Our results suggest that intraoperative OCT examination of LNs is an appropriate real-time, label-free, non-destructive alternative to frozen-section analysis, potentially offering faster interpretation and results to empower superior intraoperative decision-making.

**Conclusions:**

Intraoperative OCT has strong potential to supplement current post-operative histopathology with real-time in situ assessment of LNs to preserve both non-cancerous nodes and their lymphatic vessels, and thus reduce the associated risks and complications from surgical disruption of lymphoid structures following biopsy.

## Background

The status of lymph nodes (LNs), with or without metastatic disease, is an important factor in staging cancer, determining appropriate therapies and offering a more accurate prognosis since the transport of primary cancer cells via the lymphatic system is one of the main pathways of metastasis to distant organs. Currently, for staging breast cancer, lymph node status is predominantly evaluated via sentinel lymph node biopsy (SLNB), which involves the removal and analysis of the first, or sentinel, node(s) along the lymphatic chain of nodes draining the primary tumor [1, 2]. During breast cancer lumpectomy or mastectomy, SLNs are identified through the accumulation of a radioactive agent (Technetium-99) and/or blue dye (isosulfan or methylene) within the node, frequently resulting in the resection and submission of multiple nodes for subsequent, time-consuming, frozen-section or post-operative histopathological analysis [3–7].

While SLNB has progressively replaced axillary lymph node dissection (ALND) for the initial evaluation of nodal involvement in breast cancer staging, a recent meta-analysis of intraoperative frozen-section analysis (47 studies, 13,062 patients total) has reported a mean sensitivity of 73 % and specificity of 100 % [8]. Though this study illustrates that frozen-section has been reasonably successful in detecting *macrometastatic* disease (metastatic tumor cell foci > 2 mm) in SLNs (94 % sensitivity), *micrometastatic* disease (tumor cell foci of 0.2–2 mm) detection has proven to be evasive (40 % sensitivity), and post-operative hematoxylin and eosin (H&E) staining has shown false negative rates as high as 77 % [5–9]. Even random axillary sampling procedures and ALND limited to level 1 (lymph nodes located in the axilla) can miss metastases in 20–25 % of cases because of sampling limitations and the challenge of detecting cancer at the early stages [10, 11]. Additionally, approximately 75 % of resected nodes from all types of breast cancer are diagnosed tumor-free in post-operative permanent sections [10]. Combined with the fact that the controversial practice of delayed ALND as a second procedure after SLNB micrometastasis detection has been shown to be “oncologically meaningless in 90 % of cases” [9], it can be seen that there is an unnecessary increase of surgical cost, time and patient risk of complications, such as lymphedema and/or sensory changes due to disruption and/or obstruction of the lymphatic drainage network [5, 12, 13]. Lymphedema is considered the most significant concern due to the lifelong risk following surgery, because it occurs in 13–27 % of breast cancer surgery patients, and because it could be refractory to treatment [11]. All of these factors have fueled the ongoing debate of whether frozen-section examination in SLNB is beneficial to the patient due to the rates of surgical complications [11–18]. Thus, an intraoperative method for the in vivo assessment of SLN status that could replace frozen-section analysis by providing immediate and accurate feedback for cancer staging would thus reduce surgical complication risks by reducing the number of normal lymph nodes resected.

Current intraoperative cancer imaging or sensing techniques, such as ultrasound imaging or the detection of radioactive probes, are limited by low spatial resolution [19, 20]. These techniques, as well as x-ray computed tomography (CT), positron emission tomography (PET) and magnetic resonance imaging (MRI), can provide LN size and general morphological information, but lack the resolution to reliably detect the presence of metastatic deposits smaller than 2 mm in the LN [21–23]. In several studies, near-infrared (NIR) fluorescence imaging has been demonstrated to offer high sensitivity [24–26]. While these NIR fluorescent dye injections can be followed in real-time, the localization of the dye only shows the presence of the SLN, not whether the SLN contains metastatic disease [24–26]. Additionally, aside from ultrasound imaging, use of these screening modalities is largely disruptive to surgical workflow and prolong the procedure in much the same way as frozen-section. Therefore, the need for a superior technique to immediately, non-invasively and accurately assess LNs for the presence of metastatic disease remains.

Optical coherence tomography (OCT) provides label-free, real-time, high-resolution, microstructural imaging not possible with other imaging modalities, and is therefore better suited for the more precise localization and detection of tumor tissue [27–32]. OCT is the optical analogue of ultrasound imaging, wherein tissue is illuminated with near-infrared light and the backscattered light is collected to construct non-invasive, depth-resolved tissue images. OCT imaging resolution is roughly a factor of 10–100× higher than clinical ultrasound imaging, however OCT cannot image as deeply. The 1–2 mm imaging penetration depth (depending on the tissue type) and the cellular-level resolution of OCT enables the imaging and identification of normal LN microstructure, such as the capsule, cortex, follicles and germinal centers [30, 33–36]. Infiltration of metastatic disease in the subcapsular region of a LN and other morphological changes associated with metastatic involvement can be identified as bright, highly scattering regions (of pixels) correlated to irregularly dense, homogeneous focal areas when real-time scanning of LN tissue is performed prior to and/or immediately following surgical resection [29, 30, 33–35]. Therefore, intraoperative OCT has the potential to assess LN architecture through an intact capsule and even, without having to physically resect the LN for analysis, providing valuable and accurate real-time feedback to the surgeon on the presence or absence of metastatic disease and potentially enabling the preservation of reactive but non-metastatic nodes, reducing the risk of lymphadema.

In this study, we evaluated the sensitivity and specificity of intraoperative three-dimensional OCT (3D-OCT) for the assessment of metastatic disease in SLNs resected during breast cancer surgery, when compared to the gold-standard post-operative histopathological assessment.

## Methods

### Subject population

The study was conducted according to Declaration of Helsinki principles at two sites (University of Illinois at Urbana-Champaign and Carle Foundation Hospital) in accordance to protocols approved by the Institutional Review Boards at the participating institutions. All participants provided informed written consent prior to enrollment for both ex vivo imaging of LNs and obtaining de-identified pathology reports. Eligible patients were ages 18 years or older, diagnosed with breast cancer and scheduled for surgical intervention with sentinel/axillary LN biopsy. This study included SLNB for ductal carcinoma in situ (DCIS) cases, although the role of SLNB in DCIS cases remains controversial [9]. Patients with known infectious blood borne diseases, such as hepatitis B, hepatitis C and human immunodeficiency virus (HIV), were excluded from this study. No subject was excluded based on race, ethnic group or gender. A total of 51 subjects were imaged, but only 42 were used in post-analysis based on OCT image evaluation inclusion criteria. A summary of the patient demographics and the related tissue and tumor types is shown in Table 1.

**Table 1:** Summary of patient demographics and clinical characteristics

Characteristic	Imaged	Used	Percent
Total # of subjects	51	42	82 %
Age, years			
Mean	61.9	62.6	
Stand. Dev.	12.3	11.9	
Range	34–84	38–84	
≤ 65	29	23	
> 65	22	19	
Tumor type			
Ductal	43	35	81 %
DCIS	34	28	
IDC	36	29	
Lobular	13	12	92 %
LCIS	10	9	
ILC	10	9	
Micropapillary	5	4	80 %
Multi-type	9	8	89 %
Tumor size (greatest dimension)			
< 1 cm	15	14	93 %
1–2 cm	23	18	78 %
> 2 cm	13	10	77 %
Tissue imaged			
Lymph nodes	128	76	59 %
Imaging sites	184	99	54 %
Histopathology + nodes	118	17	14 %
Lymph node metastasis size			
(greatest dimension)			
≤ 2 mm	1	0	0 %
> 2 mm	19	17	89 %

*DCIS* ductal carcinoma in situ, *IDC* invasive ductal carcinoma, *LCIS* lobular carcinoma in situ, *ILC* invasive lobular carcinoma

### Intraoperative OCT system

**Fig. 1:**
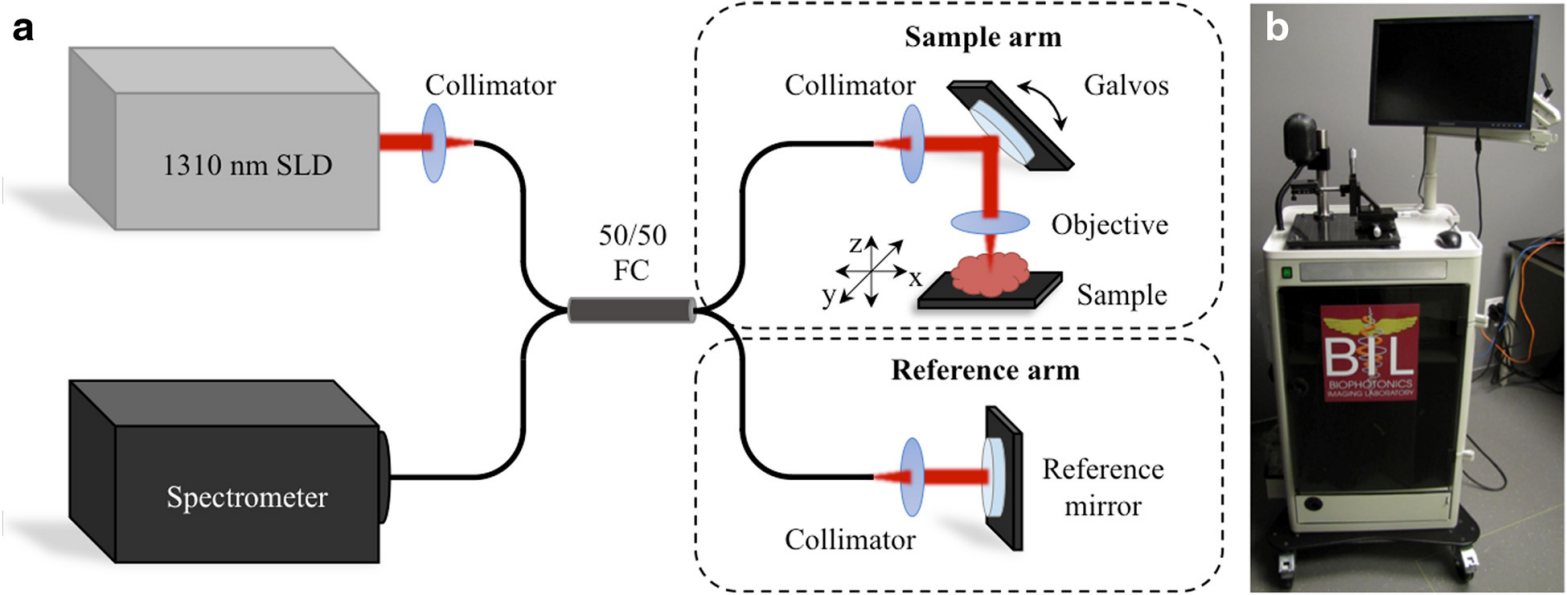
OCT system design for intraoperative assessment of ex vivo human lymph nodes for metastasis. **a** Schematic diagram and **b** photo of the OCT system. The red arrows indicate the path of near-infrared light travels along the optical fibers from the superluminescent diode (SLD) source, through the 50/50 fiber coupler (FC), splitting between the sample arm or reference arm. The reflected light from the tissue sample and the reference mirror is collected by the optical system, and travels back through the optical fibers and 50/50 FC to the line-scan detector in the spectrometer, the signal output of which was used to calculate the 3D-OCT images

The portable system used for this study included a commercial spectral domain OCT system (Bioptigen, Inc., 633 Davis Dr., Suite 480, Morrisville, NC 27560) that employed a superluminescent diode as an optical source with a center wavelength of 1310 nm. Signal from this source was passed through a 50:50 optical fiber coupler that was equally split between the reference and sample arms, shown in Fig. 1. In the sample arm, galvanometers and objective lenses (with a fixed focus) were used to volumetrically scan the tissue specimen (5 × 5 × 2 mm) at 7.4 frames/sec (6 kHz A-scan rate) via raster collection of B-scans along the x-y plane. The acquired image datasets have 11 μm axial resolution and 11 μm transverse resolution. The power at the tissue specimen was 1 mW, lower than that found in most commercially available laser pointers. Optical signal reflections collected from the sample and reference arms were recombined at the 50:50 optical fiber coupler, collimated, acquired by the spectral line camera, and stored on the system computer. All images were acquired and post-operatively reviewed with a commercial software package (InVivoVue, Bioptigen, Inc.).

### Intraoperative data acquisition

The subjects, as per standard of care, were injected with a radioactive agent (Technetium-99) and/or methylene blue dye prior to the lumpectomy or mastectomy procedure. Axillary LNs were identified by the accumulation of the injected agent and/or dye, resected individually or as an axillary contents specimen, and provided to the intraoperative research staff for 3D-OCT imaging prior to sending the tissue specimen(s) for routine histopathological examination. In a separate study, 3D-OCT imaging of ex vivo rat LNs containing these localization agents, compared to control LNs, demonstrated that the localization agents do not have a significant effect on the optical scattering or absorption properties in OCT images (data not included).

The tissue specimens were placed in sterile Petri dishes on a mounted, micrometer-positioning stage for optimizing the tissue alignment under the OCT sample arm beam to position the surface of the tissue as close to the top of the OCT imaging frame without risking image wrapping. 3D-OCT datasets were recorded from one or more locations per LN, depending on the size of the node, and marked with surgical ink, which was then set with acetic acid for subsequent correlation to histopathology. All tissue specimens were then returned to the operating room staff after no more than 10 min of OCT imaging (conducted in parallel with the ongoing surgical procedure) and sent for standard histological processing and pathological analysis. This was done under such time constraints so as to minimize impact on surgical procedure work flow, however, this did restrict the number of LNs that could be imaged during each surgery and any additional LNs resected could not be imaged and included in analysis.

### Data and statistical analysis

**Fig. 2:**
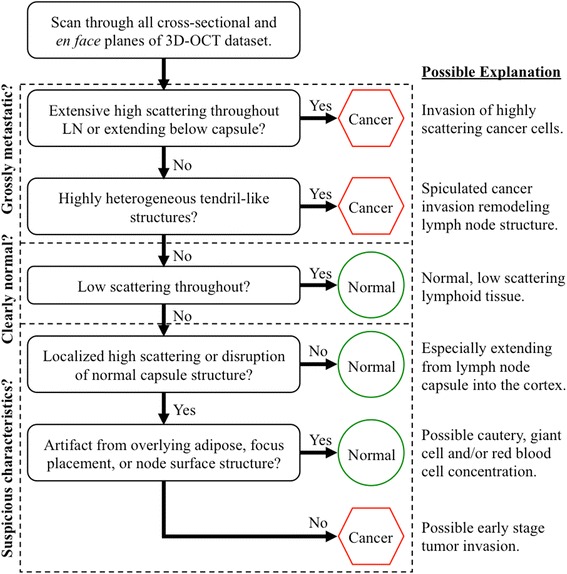
The decision tree diagram used for analyzing 3D-OCT images. A brief, sample training image set and this decision tree were developed for providing the blinded readers with direction for identifying native and abnormal lymph node anatomical structure, as well as possible image artifacts from imaging limitations in surgery. Each trained reader classified the OCT datasets as either “metastatic” or “non-metastatic”

**Fig. 3:**
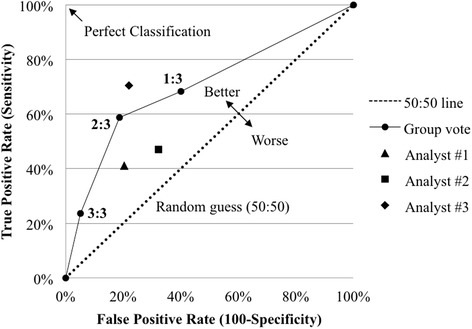
The receiver operating characteristic (ROC) curve illustrates the comparison of the true and false positive rates as the minimum “positive” vote necessary to label a lymph node metastatic. The three criteria are a single vote (1:3), majority vote (2:3) and unanimous vote (3:3). Majority voting, initially presumed, proved to be the most effective method for trained reader post-operative OCT analysis, since, of the three data points, it is the furthest from the “random guess” (50:50) line and closest to the “perfect classification” limit. Each of the individual OCT reader data are shown for comparison

We performed a double-blinded study comparing the assessment of OCT datasets to co-registered standard histopathology specimen analysis. A board-certified pathologist examined the co-registered regions on the histology slides to determine the presence of metastatic disease or other significant structural features that may be represented in the OCT image(s). Subsets of the 3D-OCT datasets that correlated with the corresponding histopathology were independently analyzed by three trained OCT readers and classified as “metastatic” or “non-metastatic”. These readers were trained using a correlated OCT-histology training set of four sample cases illustrating key OCT features of “metastatic” and “non-metastatic” cases prior to beginning the blinded analysis. Trained reader analysis of the OCT datasets was guided by the proposed decision tree, shown in Fig. 2, which was developed without input from the three readers. Datasets were not included for reader analysis if LN structure was not imaged due to limitations of OCT penetration depth and any overlying tissue obstruction, such as exceedingly thick surrounding adipose tissue (>2 mm) overlying the external capsule of the LN. The classification of individual OCT datasets was subsequently consolidated for each OCT observer. Use of a majority voting system for classifying each OCT dataset as cancerous or non-cancerous was supported by receiver operating characteristic (ROC) curve analysis (Fig. 3). Furthermore, LNs that had multiple OCT imaging locations were identified as cancerous if any of the corresponding OCT datasets were classified as cancerous. These OCT analyses for each node were then compared to histological findings, as the gold standard, to calculate system sensitivity and specificity (95 % exact, small sample confidence intervals).

## Results

### Intraoperative OCT imaging of human lymph nodes

**Fig. 4:**
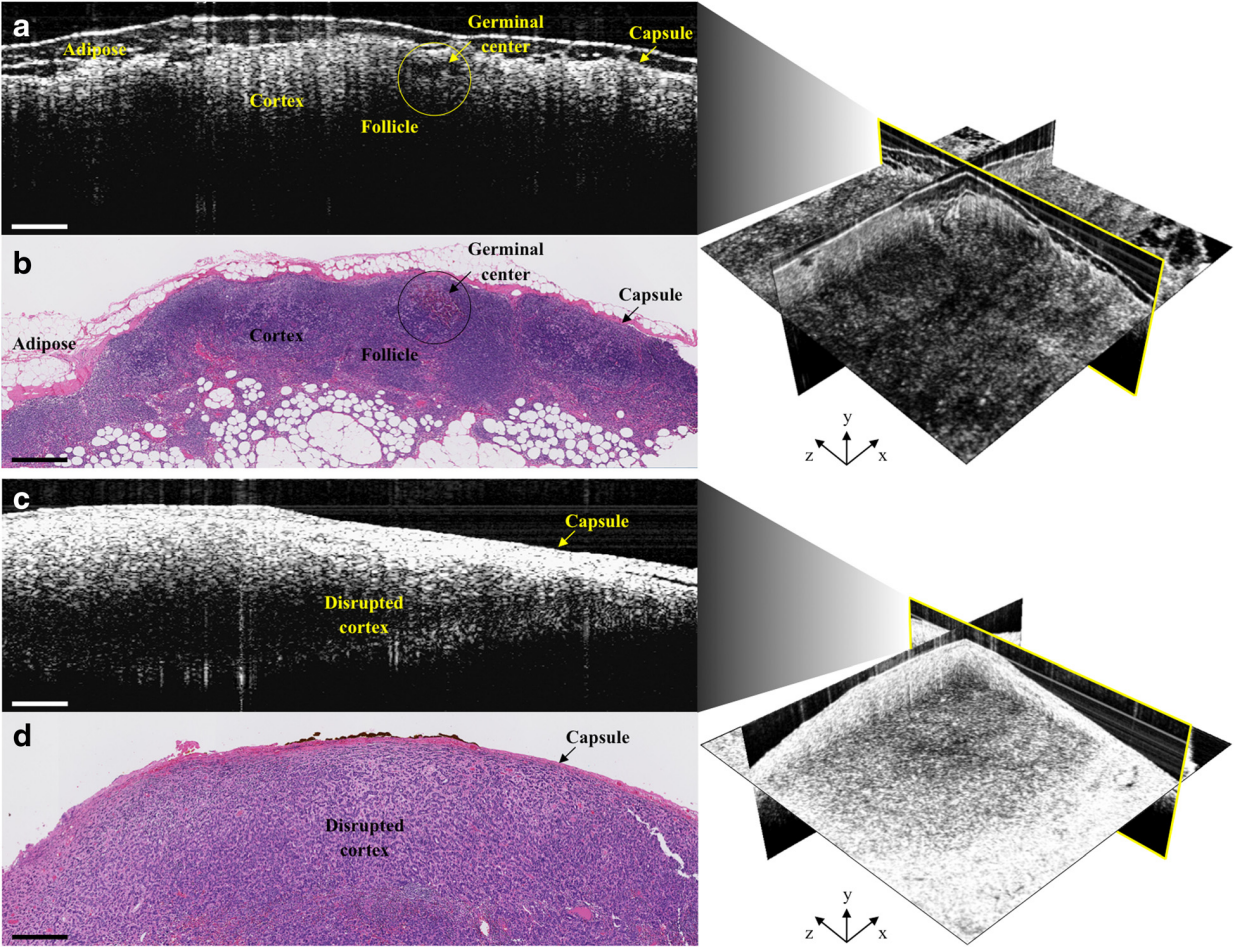
Representative intraoperative OCT (**a** & **c**) and corresponding histopathology (**b** & **d**) images of a normal, non-metastatic (*top*) and cancerous, metastatic (*bottom*) human lymph node. In **a** & **b**, normal lymph node structures, such as the capsule, cortex, follicles and germinal centers, as well as adipose, can be identified in both images. In **c** & **d**, metastatic invasion of cancer cells disrupts the normal lymph node cortex structure and can disrupt identification of follicles and germinal centers. All scale bars: 0.5 mm

**Fig. 5:**
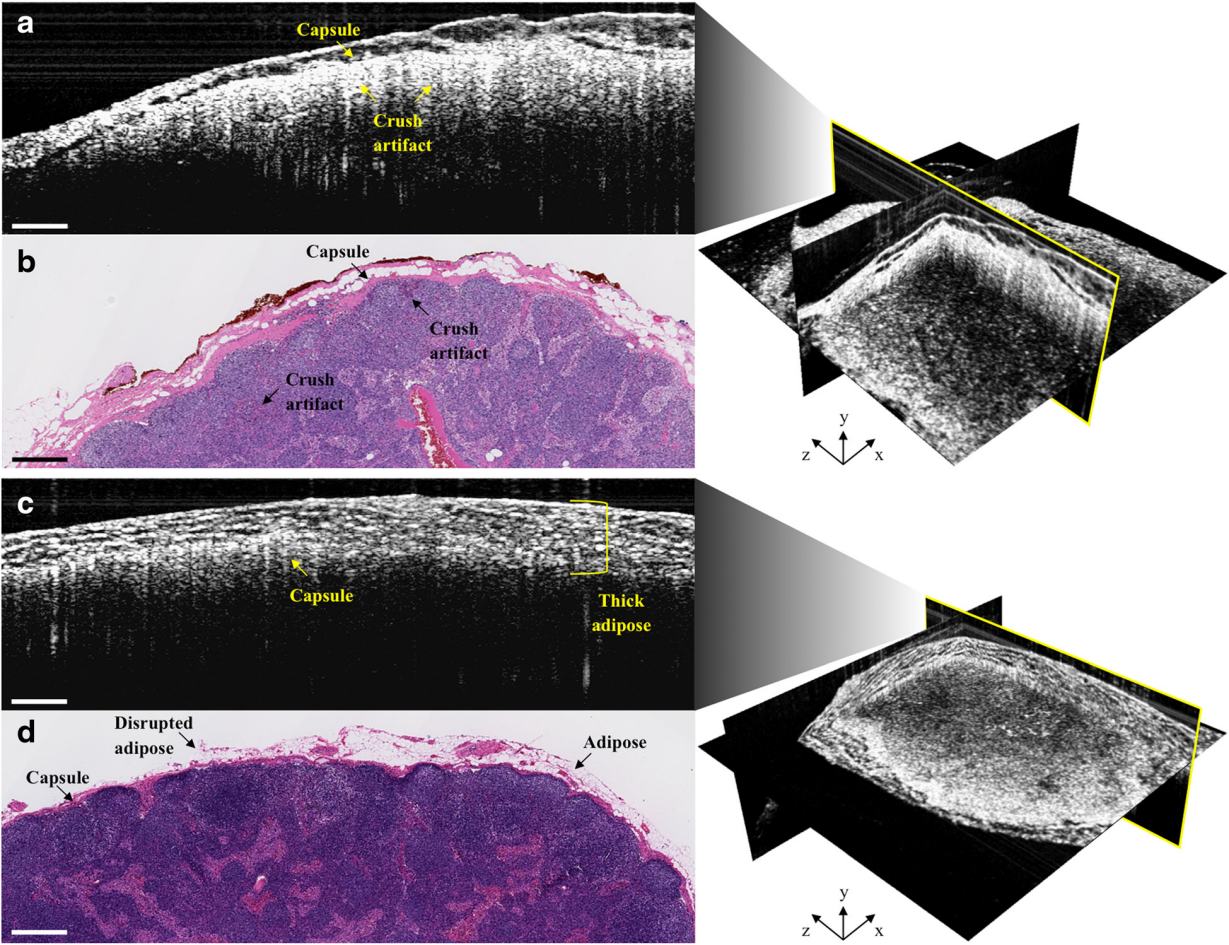
Representative intraoperative OCT (**a** & **c**) and corresponding histopathology (**b** & **d**) images of false positive (*top*) and false negative (*bottom*) cases. False positives can result from crush artifact, which mimics the bright white (*high*) OCT signal intensity of metastatic cancer cell invasion (Fig. 4c). False negatives can result from thick overlying adipose, which reduces imaging depth penetration, affects optical beam quality and resolution, and, consequently, underlying lymph node signal intensity. Some of the overlying adipose in the histology (**d**) is missing, most likely disrupted and/or lost during tissue processing. All scale bars: 0.5 mm

Our mobile cart-based intraoperative OCT system was utilized in 51 operations for patients with a preoperative diagnosis of carcinoma, wherein 184 3D-OCT image sets from 128 LNs were acquired immediately following surgical resection and prior to submission of the specimens for standard histopathological processing and analysis (Table 1). The ex vivo imaging sessions required between 5 and 10 min, depending on the size and number of tissue specimens, as well as the number of images acquired, comparatively a fraction of the time required for frozen-section processing and analysis. While most operations had fewer than 5 LNs resected and every LN was able to be imaged within the time constraint, there were several cases where 15–53 LNs were resected. During these cases, only a fraction of the LNs could be imaged and included in the study, without disrupting the surgical procedure workflow. Only 99 3D-OCT images of 76 lymph nodes from 42 operations were used in post-analysis, which, along with surgical imaging time constraints, was based on OCT image evaluation inclusion criteria, namely if the lymph node tissue was present in the correlated histology and OCT image region(s). In the other cases, LN tissue was absent from the OCT images due to excess overlying adipose tissue. Representative correlated cases of a normal non-metastatic human LN and a cancerous metastatic human LN are shown in Fig. 4. In comparison, representative correlated images of a false positive and a false negative case are shown in Fig. 5.

### Decision tree-guided lymph node OCT assessment

Similar to image-based representations in x-ray mammography, bright white regions in the OCT images indicate dense, highly scattering tissue potentially indicative of metastatic disease. Accordingly, a brief, sample training image set and decision tree (Fig. 2) were developed, from knowledge attained from prior work [30, 33–35]. This provided the three OCT-experienced readers with direction for identifying native LN anatomical structure, irregular highly scattering tissue localization and extension, and possible image artifacts from imaging limitations in surgery, such as too much overlying adipose or dense tissue affecting imaging penetration. Ultimately, each trained reader classified the LN OCT datasets as “metastatic” or “non-metastatic”. A majority voting system (2 “metastatic” classifications) was initially anticipated to be the most effective assessment method.

An ROC curve, a standard graphical means for analyzing a binary classifier system (i.e. metastatic vs. non-metastatic) [37], was utilized to visually validate which of the following methods was most effective for classifying LNs under the presented trained reader, post-operative OCT analysis: single “metastatic” reading from a trained OCT reader, majority vote or unanimous decision. Each individual reader’s sensitivity and specificity are also indicated and there is no statistically significant difference between individual readers when comparing each reader’s confidence interval. Majority voting, which is not unlike other decision-making procedures in diagnostics, was verified (Fig. 3) to be the most effective assessment method because it is the point quantifiably illustrated as the furthest from the “random guess” line and closest to the “perfect classification” point.

From majority voting OCT reader analysis, sensitivity and specificity were 58.8 % (95 % CI, 32.9–81.6) and 81.4 % (95 % CI, 69.1–90.3), respectively. Considered in other terms, hypothetically if OCT imaging were performed in situ with a hand-held surgical imaging probe, and OCT was used alone to guide LN resection in all of these cases without the use of an injectable tracer or palpation information, the procedures would have resulted in a total of only 21 resected LNs, instead of 76, over 42 surgeries. This would have reduced the total number of resected LNs by 72 % by preventing the unnecessary resection of 48 non-metastatic LNs, but forgoing the resection of 7 metastatic LNs. In this study, the confidence intervals for sensitivity are wide due to a limited number of positive (“metastatic”) sites, while the confidence intervals for specificity are narrower due to a larger number of negative (“non-metastatic”) sites imaged. The percentage of resected, positive (“metastatic”) LNs in our study was originally in accordance with the national standard (less than 10 %) [5, 11, 13]. Toward the end of the study, however, we specifically recruited pre-operative biopsy-proven positive cases to increase our proportion of positive LNs to 22 % in an attempt to develop a more balanced statistical analysis.

## Discussion

These results demonstrate that OCT has the potential to be an informative and rapid imaging modality for intraoperative assessment and surgical decision-making of metastatic disease in human lymph nodes. The high specificity indicates a low number of false positives (Fig. 5a and b), which means that when the LN is “non-metastatic”, our readers were more readily able to identify normal LN tissue (Fig. 4a and b). This could potentially enable the identification and reduced resection of truly non-reactive, normal LNs and thereby reduce the incidence of lymphedema. However, the moderate sensitivity indicates a poor number of false negatives (Fig. 5c and d), or when the LN is “metastatic”, our readers misidentified a cancerous LN for a non-cancerous one. This is in large part due to image artifacts that were also present in many prior ex vivo OCT imaging studies, and though this information was used to develop the brief, sample training image set and decision tree (Fig. 2), reader analysis difficulty persists [30, 33–35]. In some of these cases, the OCT signal intensity from a cancerous region(s) was shadowed or reduced by overlying, highly dense stromal tissue, producing a strong OCT signal reflection with diminished signal from deeper tissue (Fig. 5c and d). Additionally, in some cases, thick, overlying adipose tissue restricted the cancerous region in the middle of the LN to the bottom half of the OCT frame, where signal intensity is again weaker from scattering effects, refraction and shadowing, illustrated in Fig. 5c and d. Furthermore, in several cases, the overlying adipose layer was disrupted and/or lost due to its inherent fragility during histopathological processing. This can make correlation with OCT more difficult since the tissue marking inks can consequently be disrupted and/or lost during processing, constraining correlations to be made solely through matching LN morphology (Fig. 5d). While real-time, OCT-guided LN evaluation is non-destructive and less time consuming than frozen-section analysis, a frozen-section preparation provides deeper tissue visualization. Although the imaging team was restricted by our IRB protocol from doing so in this study, future intraoperative OCT use might require the removal or displacement of any overlying adipose tissue to better expose the LN capsule, maximize OCT imaging depth into the node, and improve overall signal quality through reduced scattering, refraction and shadowing artifacts. For suspicious and/or large tissue samples, hemisection followed by OCT imaging of the corresponding cut surfaces would enable imaging and visualization of deeper LN structures comparable to standard frozen-section analysis, while still remaining the faster of the two techniques.

Interestingly, in all false positive 3D-OCT datasets, the corresponding histology revealed crush artifacts and/or grossly reactive, yet histologically non-metastatic, nodules (Fig. 5b), which are much more dense than normal LN tissue and could produce higher optical scattering similar to that from metastatic cell infiltration (Fig. 4c). The crush artifacts could also be attributed to tissue trauma during resection or gross histopathological sectioning, and therefore in situ imaging could reduce the occurrence of these artifacts in the OCT images. Given that the current surgical standard supports resection of suspicious LNs, OCT may offer the most surgical benefit by providing additional means for identifying normal LNs and thereby reduce unnecessary resection of additional LNs beyond the true SLNs, thereby reducing unnecessary surgical disruption of lymphoid tissue and associated lymphatic vessels.

Further improvements in OCT resolution, through the incorporation of computed imaging techniques such as interferometric synthetic aperture microscopy (ISAM) [38, 39] and computational adaptive optics (CAO) [40, 41] could further enhance the real-time diagnostic capabilities of OCT in the operating room. Use of injectable magnetic nanoparticles [42], such as iron oxide, has shown promise as an alternative to the current radiotracers or dyes, as well as a method for improving OCT image quality and cancer detection when coupled with magnetomotive OCT [43]. Optical needle probes used for beam-delivery in OCT systems could be inserted into suspicious in vivo LNs to provide greater OCT imaging depth penetration while minimizing tissue disruption, especially compared to LN hemisection [44–46]. Additionally, improvements in OCT data acquisition rates could facilitate faster intraoperative imaging of all exposed tissue surfaces, whether in vivo or across an ex vivo specimen, to construct a more complete evaluation, especially when coupled with ISAM and CAO [47]. These techniques could be integrated into surgical systems to improve OCT depth resolution and acquisition speed in real-time, and enable further improved detection of metastases not possible with current medical imaging technology. Further improvements and coupling with optical molecular imaging techniques like nonlinear interferometric vibrational imaging (NIVI) of intrinsic molecular markers could produce a more sensitive and accurate color-coded image differentiating cancer from normal tissues [48].

Although all LN metastases evaluated in this study were classified as macrometastases, ongoing work with an intraoperative handheld OCT probe functioning with near-cellular resolution could lead to identification of LN metastases smaller than 2 mm. Additionally, future intraoperative OCT-guided assessment of in situ LNs by the surgeon will incorporate additional key information that will contribute towards diagnosis. Although there are limitations to OCT, such as under-sampling while imaging, and the extended time needed for scanning/sampling an entire node, these limitations are also present in frozen-section analysis. Furthermore, while our trained readers were blinded during their assessment of only the 3D-OCT datasets, intraoperative surgical OCT-guided assessment will be accompanied by far more subject case and procedural knowledge, such as pre-operative findings, LN size, visual and palpable features and the presence of injectable radiotracer and/or dye. Therefore, future studies incorporating intraoperative in vivo 3D-OCT assessment by surgeons will likely facilitate a more practical evaluation of OCT compared to frozen-section analysis. The potential role for a surgical pathologist to review and interpret the real-time 3D-OCT data during the surgical procedure also exists, as an alternative to their role in sectioning, staining and interpreting frozen-section histopathology. While real-time, 3D-OCT transcapsule imaging will not likely replace surgical specimen histopathology (where true cellular and molecular diagnostics are achieved), it does enable advantages for both assessment speed and in vivo imaging, which are preferred over any ex vivo technique when considering tissue conservation. Use of an intraoperative handheld probe for in vivo assessment of breast cancer [49] could also enable LN evaluation prior to removal, and a potential corresponding reduction of risk of complications such as lymphedema. Therefore, OCT could provide a more effective means for LN assessment and intraoperative cancer staging while potentially reducing unnecessary surgery time and the time and costs associated with extensive frozen-section or post-operative histopathology for the large number of histologically normal LNs.

## Conclusion

Current intraoperative cancer imaging and sensing techniques are limited by low spatial resolution and a clear need remains for a superior technique that can non-invasively and accurately assess LNs for the presence of metastatic disease. Intraoperative OCT has strong potential to supplement current post-operative histopathology with real-time in situ assessment of LNs to preserve both non-cancerous nodes and their lymphatic vessels. Use of such technology could reduce the associated risks and complications from surgical disruption of lymphoid structures following biopsy.

## Abbreviations

ALND: axillary lymph node dissection
CAO: computational adaptive optics
CI: confidence interval
CT: x-ray computed tomography
DCIS: ductal carcinoma in situ
FC: fiber coupler
H&E: hematoxylin and eosin
HIV: human immunodeficiency virus
IDC: invasive ductal carcinoma
ILC: invasive lobular carcinoma
ISAM: interferometric synthetic aperture microscopy
LCIS: lobular carcinoma in situ
LN: lymph node
MRI: magnetic resonance imaging
NIR: near-infrared
NIVI: nonlinear interferometric vibrational imaging
OCT: optical coherence tomography
PET: positron emission tomography
ROC: receiver operating characteristic
SLD: superluminescent diode
SLNB: sentinel lymph node biopsy
UIUC: University of Illinois at Urbana-Champaign
